# Incisional hernia repair by synthetic mesh prosthesis in patients with inflammatory bowel disease: a comparative analysis

**DOI:** 10.1186/s12893-021-01350-9

**Published:** 2021-09-27

**Authors:** Daniel Heise, Charles Schram, Roman Eickhoff, Jan Bednarsch, Marius Helmedag, Sophia M. Schmitz, Andreas Kroh, Christian Daniel Klink, Ulf Peter Neumann, Andreas Lambertz

**Affiliations:** Department of General, Visceral and Transplantation Surgery, University Hospital RWTH Aachen, Pauwelsstr. 30, 52074 Aachen, Germany

**Keywords:** Incisional hernia, Inflammatory Bowel Disease, Mesh prothesis, Morbidity, Postoperative outcome

## Abstract

**Background:**

Patients with inflammatory bowel disease (IBD) have a high-life time risk undergoing abdominal surgery and are prone to develop incisional hernias (IH) in the postoperative course. Therefore, we investigated the role of IBD as perioperative risk factor in open ventral hernia repair (OVHR) as well as the impact of IBD on hernia recurrence during postoperative follow-up.

**Methods:**

The postoperative course of 223 patients (Non-IBD (n = 199) and IBD (n = 34)) who underwent OVHR were compared by means of extensive group comparisons and binary logistic regressions. Hernia recurrence was investigated in the IBD group according to the Kaplan–Meier method and risk factors for recurrence determined by Cox regressions.

**Results:**

General complications (≥ Clavien-Dindo I) occurred in 30.9% (72/233) and major complications (≥ Clavien-Dindo IIIb) in 7.7% (18/233) of the overall cohort with IBD being the single independent risk-factor for major complications (OR = 4.2, p = 0.007). Further, IBD patients displayed a recurrence rate of 26.5% (9/34) after a median follow-up of 36 months. Multivariable analysis revealed higher rates of recurrence in patients with ulcerative colitis (UC, 8/15, HR = 11.7) compared to patients with Crohn’s disease (CD, 1/19, HR = 1.0, p = 0.021).

**Conclusion:**

IBD is a significant risk factor for major postoperative morbidity after OVHR. In addition, individuals with IBD show high rates of hernia recurrence over time with UC patients being more prone to recurrence than patients with CD.

## Introduction

Incisional hernias (IH) are a common complication after abdominal surgery with an incidence of up to 20% [1]. A history of multiple laparotomies, immunosuppressive therapy or any degree of malnutrition are known risk factors for the development of IH over time [2, 3]. Therefore, patients with inflammatory bowel disease (IBD) are at high risk for the development of IH as these individuals usually display a variety of risk factors. The high prevalence of surgery in IBD patients with 70% of patients with Crohn's disease (CD) and 35% of patients with Ulcerative colitis (UC) require abdominal surgery during the course of their disease does further underline the importance of IH in IBD patients [4]. In these complex situations, an open ventral hernia repair (OVHR) with mesh augmentation is usually required.

While a variety of studies have already examined the efficacy of this technique, there are only a few studies focusing on IBD patients with heterogeneous patient cohorts over long periods of time [5, 6]. Furthermore, various techniques with partly biological and partly synthetic mesh implants have also been included in some of the studies [2]. This limited data within in the literature allows only a limited analysis of the long-term outcome after IH repair in IBD patients. It also remains to be determined whether patients with IBD are subject to a higher perioperative risk in IH repair compared to non-IBD patients, especially concerning implantation of a synthetic mesh prosthesis. The purpose of this study was subsequently to analyze the short-term results of IH repair in IBD in contrast to non-IBD patients at tertiary referral center for the surgical treatment of IH. Additionally, we further aimed to determine the long-term outcome of OVHR in IBD patients and to identify risk factors that are associated with hernia recurrence.

## Patients and methods

We here report a retrospective analysis evaluating postoperative outcome after IH repair in patients with or without IBD at the Department of Surgery and Transplantation at University Hospital Aachen, Germany. Therefore, we studied 652 patients who underwent OVHR with sublay mesh augmentation at our institution between January 2005 and March 2018. Approval by the Ethics-committee of the medical faculty, RWTH University, Aachen, Germany (EK 090/18) was obtained before analysis of the data. All methods were performed in accordance with the relevant guidelines and regulations. The need for patients informed written consent was waived due to the retrospective nature of the study. No patient data were collected in addition to guideline-required therapy. Exclusion criteria were an additional transversal laparotomy, IH < 200 cm^2^, parastomal hernia, laparoscopic repair and missing clinical data. A total of 233 patients, who underwent IH were finally included in the analysis.

### *Preoperative workup*

The indication for surgery was determined by a specialized surgeon. IH was defined as the development of an abdominal wall defect in a midline incision. All assigned patients were preoperatively examined in detail. Transabdominal ultrasound and/or contrast-material enhanced computed tomography was performed to assess the extent of the IH and to exclude potential additional fascial defects. Other variables recorded include age, sex, body mass index (BMI), The American Society of Anesthesiologists (ASA) score, and comorbidities.

### Surgical technique and postoperative management

IH repair was performed as OVHR with mesh augmentation in sublay position. After excision of the skin scar the abdominal cavity was opened. Then, the rectal sheath was dissected after intestinal adhesiolysis to establish a retromuscular mesh depot with an overlap of at least 5 cm in all directions. A PVDF-mesh (DynaMesh®, FEG-Textiltechnik, Aachen, Germany) was placed in sublay position on the peritoneum and posterior rectus sheath. Finally, an absorbable running suture was used to reconstruct the anterior fascia. Redon drains were positioned on the mesh and in case of a large wound surface, additionally subcutaneously. Postoperatively, patients were monitored in the intensive care unit depending on previous diseases and size of the hernia. With regard to diet build-up, patients were allowed to drink clear liquids on the day of surgery. If this was well tolerated, a gradual early food intake was provided. Depending on their clinical status, patients were intensively mobilized early and provided with respiratory therapy. A laboratory test was performed on the first postoperative day. The first dressing change with extraction of any intraoperative drains was performed on the second postoperative day. Discharge was sought on the fourth to fifth postoperative day in the absence of contraindications. A sonographic examination was performed on the discharge day, and patients continued to be seen in the outpatient clinic after 7–10 days for clinical assessment and ultrasound follow-up.

### Data collection

All study data including demographics, clinical characteristics, preoperative treatment with steroids and/or immunosuppressive therapy within 6 weeks of surgery and operative and postoperative data of every patient was prospectively collected within an institutional database. The postoperative course was reviewed for complications and rated according to the Clavien-Dindo classification. Follow-up was performed for all IBD patients in our outpatient clinic or by the patient's gastroenterologist. Patients with clinical symptoms or suspected recurrence were presented to a specialized hernia outpatient clinic in the surgical department, where they were examined for recurrence by ultrasound. There were no differences between Cohn`s disease and Ulcerative colitis with regard to postoperative management. Recurrence-free survival was defined as the interval between the date of OVHR and the date of recurrence or last follow-up in patients without recurrence. Patients were monitored until September 2018.

### Statistical analysis

The primary endpoint of this study was the occurrence of postoperative complications in IBD patients compared to non-IBD patients undergoing OVHR. Additionally, univariate and multivariable analyses of the whole cohort were performed to identify risk factors for the occurrence of overall (Clavien-Dindo ≥ 1) and major (Clavien-Dindo ≥ 3b) complications. Categorical data are presented as counts and percentages and compared using the chi-square test. Data derived from continuous variables are presented as mean and standard deviation and are analyzed by the Mann–Whitney *U* test. Associations between perioperative variables and complications were assessed by means of binary logistic regression. ASA, BMI, age and variables being statistically significant in univariate analysis were transferred into a multivariable model and analyzed with multivariable binary logistic regressions. For this purpose, nominal and categorial data were recoded into scaled dummy variables.

A further subgroup analysis of hernia recurrence in the IBD-group was done using the Kaplan–Meier method. The median follow-up was 36 months. The results were plotted in Kaplan–Meier curves and compared using the log-rank test. Cox regression analysis was applied to examine the impact of the clinical and perioperative variables on hernia recurrence. Variables associated with hernia recurrence with a p-value less than 0.1 in a univariate proportional hazards model were subsequently entered into a Cox multivariate regression model with subsequent backward elimination. The level of significance was set to p < 0.05, and p values are given for two-sided testing. Analyses were performed using SPSS Statistics 25 (IBM Corp., Armonk, NY, USA).

## Results

We here analyzed a cohort of 233 patients who underwent OVHR between January 2005 and March 2018 at the Department of Surgery and Transplantation at University Hospital Aachen, Germany. Table 1 shows the clinical data and perioperative characteristics of Non-IBD (n = 199) and IBD (n = 34) patients, respectively. We observed no significant differences between the groups regarding preoperative characteristics except for a higher BMI in the Non-IBD group (Non-IBD: 29.0 ± 5.9 kg/m^2^ vs. IBD: 27.0 ± 5.5 kg/m^2^; p = 0.043). An Analysis of the perioperative data revealed a higher rate of intraoperative blood transfusions (Non-IBD: 1 (0.5%) vs. IBD: 3 (8.8%); p = 0.001), major complications (Non-IBD: 11 (5.5%) vs. IBD: 7 (20.6%); p = 0.001), and postoperative relaparotomies (Non-IBD: 7 (3.5%) vs. IBD: 5 (14.7%); p = 0.006) in the IBD group.

**Table 1:** Clinical and perioperative characteristics of Non-IBD and IBD patients (*n* = 233)

	Non-IBD (*n* = 199)	IBD (*n* = 34)	*p*-value	Total (*n* = 233)
Demographics				
Sex, n (%)			.926	
Male	133 (66.8)	23 (67.6)		156 (67.0)
Female	66 (33.2)	11 (32.4)		77 (33.0)
Age (years)	59.5 ± 13.5	61.9 ± 13.1	.507	59.9 ± 13.5
BMI (kg/m^2^)	29.0 ± 5.9	27.0 ± 5.5	.043	28.7 ± 5.8
ASA, n (%)			.760	
I	2 (1.0)	0 (0)		2 (0.0)
II	119 (59.8)	22 (64.7)		141 (60.5)
III	74 (37.2)	12 (35.3)		86 (36.9)
IV	4 (2.0)	0 (0)		4 (1.7)
V	0 (0)	0 (0)		0 (0)
CRD, n (%)	16 (8)	7 (20.6)	.054	23 (9.9)
DM, n (%)	40 (20.1)	2 (5.9)	.053	42 (18.0)
CVD, n (%)	31 (15.6)	10 (29.4)	.084	41 (17.6)
CKD, n (%)	27 (13.6)	3 (8.8)	.445	30 (12.9)
History of malignancy, n (%)	49 (24.6)	6 (17.6)	.376	55 (23.6)
Perioperative data				
Intraoperative blood transfusion, n(%)	1 (0.5)	3 (8.8)	.001	4 (1.7)
ICU-stay, n(%)	24 (12.1)	8 (23.5)	.073	32 (13.7)
Postoperative complications, n (%)			.004	
No complications	141 (70.9)	18 (56.3)		159 (68.8)
Clavien-Dindo I	5 (2.5)	2 (6.3)		7 (3.0)
Clavien-Dindo II	11 (5.5)	1 (3.1)		12 (5.2)
Clavien-Dindo IIIa	31 (15.6)	4 (12.5)		35 (15.2)
Clavien-Dindo IIIb	8 (4.0)	6 (18.8)		14 (6.1)
Clavien-Dindo IVa	0 (0)	1 (3.1)		1 (0.4)
Clavien-Dindo IVb	0 (0)	0 (0)		0 (0)
Clavien-Dindo V	3 (1.5)	0 (0)		3 (1.3)
≥ Clavien-Dindo I	58 (29.1)	14 (41.2)	.161	72 (30.9)
≥ Clavien-Dindo IIIb	11 (5.5)	7 (20.6)	.002	18 (7.7)
SSI	12 (6.0)	5 (14.7)	.072	17 (7.3)
Seroma	33 (16.7)	9 (26.5)	.166	42 (18.0)
Relaparotomy	7 (3.5)	5 (14.7)	.006	12 (5.2)

Data presented as median and standard deviation if not noted otherwise. Categorical data were compared using the chi-squared test, fisher’s exact test or linear-by-linear association according to scale and number of cases. Data derived from continuous variables of different groups were compared by Mann–Whitney-*U*-Test. *BMI* body mass index, *ASA* American society of anesthesiologists classification, *CRD* chronic respiratory disease, *DM* diabetes mellitus, *CVD* cardiovascular disease, *CKD* chronic kidney disease, *ICU* intensive care unit; SSI, surgical site infection

### Uni- and multivariable analysis of postoperative morbidity

For a more detailed assessment of the postoperative morbidity a univariate binary logistic regression analysis was carried out (Table 2). In our cohort, the necessity of an intensive care stay (HR = 3.5; p = 0.001) was associated with the occurrence of any postoperative complication (Clavien Dindo ≥ 1). We subsequently included all variables with p < 0.1 in a multivariable binary logistic regression model which determined a history of malignancy (HR = 2.14; p = 0.045) and also the necessity of an intensive care stay (HR = 3.67; p = 0.001) as significant predictors of postoperative morbidity (Table 3). A complementary analysis on major postoperative morbidity (Clavien-Dindo ≥ 3b) was also carried out. Univariable analysis showed a significant association of present IBD (HR = 4.43; p = 0.005) and intraoperative blood transfusion (HR = 13.31; p = 0.012) with major postoperative complications. These variables were also included in the corresponding multivariable binary logistic regression model which identified the presence of IBD (HR = 4.19; p = 0.007) as the single independent predictor of major postoperative morbidity (Table 4).

**Table 2:** Univariable analysis of perioperative morbidity in Non-IBD and IBD patients (n = 233)

	n (%)	Major morbidity (Clavien Dindo ≥ IIIb)			Morbidity (Clavien Dindo ≥ I)		
		Hazard ratio	95% CI	*P* value	Harzard ratio	95% CI	*P* value
Gender				.283			.506
Male	156 (67.0)						
Female	77 (33.0)						
Age				.611			.372
≤ 60	116 (49.8)						
> 60	117 (50.2)						
BMI				.626			.061
≤ 30	154 (66.1)						
> 30	61 (38.6)						
ASA				.632			.813
≤ II	143 (61.4)						
> II	90 (38.6)						
CVD				.458			.217
Present	41 (17.6)						
Absent	192 (82.4)						
CKD				.816			.591
Present	30 (12.9)						
Absent	203 (87.1)						
DM				.998			.994
Present	42 (18.0)						
Absent	191 (82.0)						
CRD				.322			.371
Present	23 (9.9)						
Absent	210 (90.1)						
History of malignancy				.474			.098
Present	55 (23.6)						
Absent	178 (76.4)						
IBD				.005			.164
Present	34 (14.6)	4.43	1.58–12.41				
Absent	199 (85.4)	1					
Intraoperative blood transfusion				.012			.999
Yes	4 (1.7)	13.31	1.76–100.83				
No	229 (98.3)	1					
Intensive care				.283			.001
Yes	32 (13.7)				3.50	1.63–7.52	
No	201 (86.3)						

Various parameters are associated with major and general postoperative morbidity. Hazard ratios are shown for statistically significant variables. *ASA* American society of anesthesiologists classification, *BMI* body mass index, *CVD* cardiovascular disease, *CKD* chronic kidney disease, *DM* diabetes mellitus, *CRD* chronic respiratory disease, *IBD* inflammatory bowel disease

**Table 3:** Multivariable binary logistic regression of perioperative morbidity in Non-IBD and IBD patients (n = 233)

Variable	Morbidity (Clavien Dindo ≥ I)		
	Hazard ratio	95% CI	*P* value
Age			.433
≤ 60			
> 60			
BMI			.128
≤ 30			
> 30			
ASA			.433
≤ II			
> II			
History of malignancy			.045
Present	2.14	1.08–4.52	
Absent	1		
Intensive care			.001
Yes	3.67	1.65–8.15	
No	1		

All variables showing p < 0.1 in univariate binary logistic regression were included in a multivariable logistic regression. Hazard ratios are shown for statistically significant variables. *ASA* American society of anesthesiologist’s classification, *BMI* body mass index

**Table 4:** Multivariable binary logistic regression of major perioperative morbidity in Non-IBD and IBD patients (n = 233)

Variable	*Major morbidity (Clavien Dindo* ≥ *IIIb)*		
	Hazard ratio	95% CI	*P* value
Age			.479
≤ 60			
> 60			
BMI			.535
≤ 30			
> 30			
ASA			.621
≤ II			
> II			
IBD			.007
Present	4.185	1.49–11.77	
Absent	1		
Intraoperative blood transfusion			.069
Yes			
No			

All variables showing p < 0.1 in univariate binary logistic regression were included in a multivariable logistic regression. Hazard ratios are shown for statistically significant variables. *ASA* American society of anesthesiologist’s classification, *BMI* body mass index, *IBD* inflammatory bowel disease

### Hernia recurrence in IBD subgroup

**Fig. 1:**
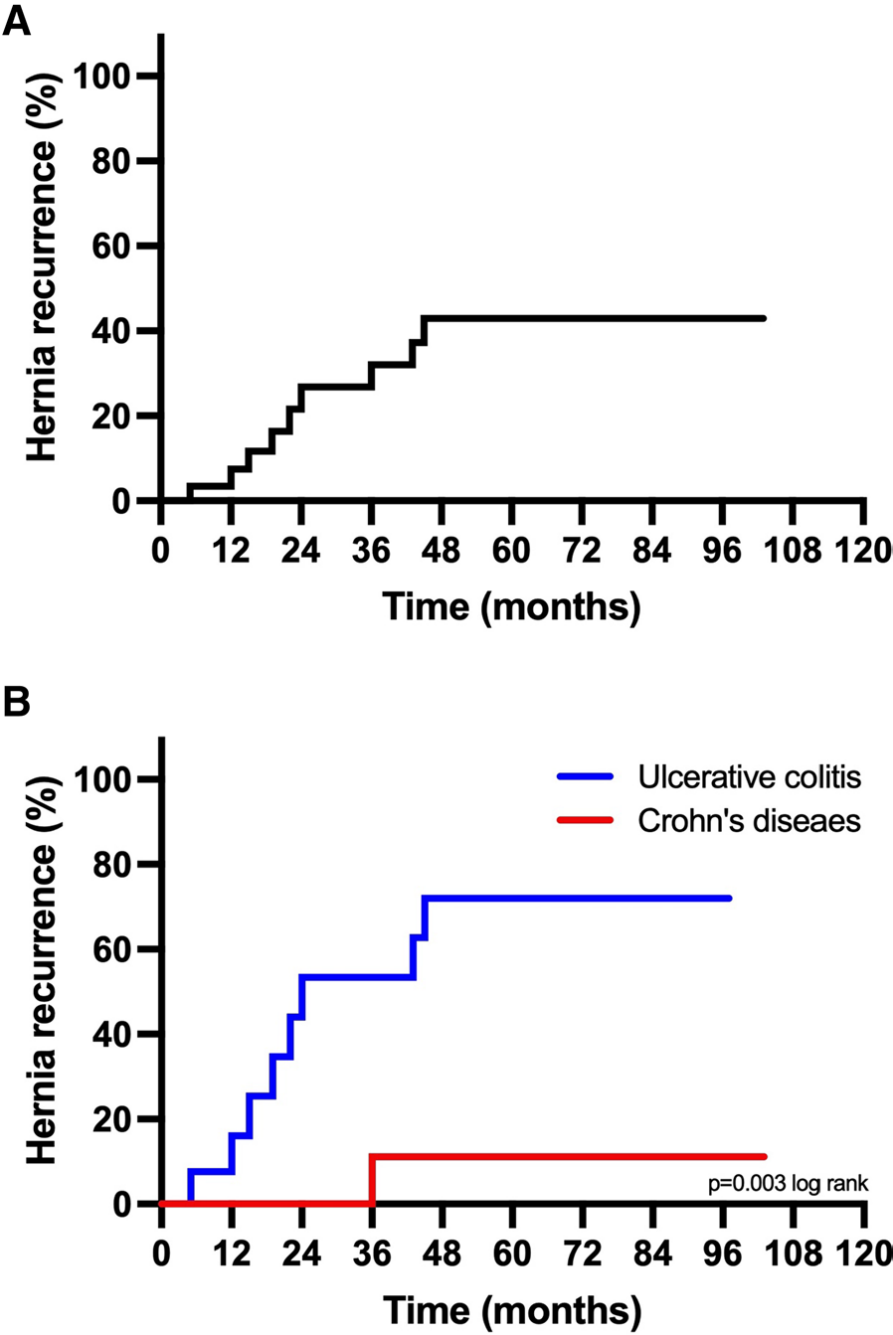
Kaplan–Meier Curve of hernia recurrence in IBD subgroup (**A**) and UC vs. CD (**B**)

After a median follow up of 36 months, hernia recurrence was observed in 9 out of 34 IBD patients (26.5%). Figure 1a shows the Kaplan–Meier Curve for the time to recurrence. In the IBD subgroup 15 patients (44.1%) presented with ulcerative colitis (UC) and 19 (55.9%) with Crohn’s disease (CD). Independent prognostic factors were evaluated using the Cox regression proportional hazard model. Univariable analysis showed a significant association of UC (HR = 11.68; p = 0.021), history of > 1 bowel resection, and extraintestinal manifestation (HR = 13.31; p = 0.012) with the occurrence of a recurrent hernia. All variables with p < 0.1 were also included in the corresponding multivariable Cox regression model which identified the presence of UC (HR = 11.68; p = 0.021) as the independent predictor of hernia recurrence as shown in Table 5. Using the log rank test as a score test for the Cox regression model UC was also identified as more frequently associated with IH recurrence compared to CD (p = 0.003) as shown in Fig. 1b.

**Table 5:** Univariable and multivariable analysis of hernia recurrence in IBD patients (n = 34)

	n (%)	Univariable analysis			Multivariable analysis		
		Hazard ratio	95% CI	*P* value	Harzard ratio	95% CI	*P* value
Gender				.079			.564
Male	23 (67.6)						
Female	11 (32.4)						
Age				.756			
≤ 60	17 (50.0)						
> 60	17 (50.0)						
BMI				.233			
≤ 30	25 (73.5)						
> 30	9 (26.5)						
ASA				.054			.266
≤ II	22 (64.7)						
> II	12 (35.3)						
CVD				.612			
Present	10 (29.4)						
Absent	24 (70.6)						
CKD				.606			
Present	3 (8.8)						
Absent	31 (91.2)						
DM				.593			
Present	2 (5.9)						
Absent	32 (94.1)						
CRD				.441			
Present	7 (20.6)						
Absent	27 (79.4)						
History of malignancy				.358			
Present	6 (17.6)						
Absent	28 (82.4)						
Type of IBD				.021			.021
Ulcerative colitis	15 (44.1)	11.68	1.45–93.57		11.68	1.45–93.57	
Crohn`s disease	19 (55.9)	1			1		
History of > 1 bowel resection				.035			.096
Yes	8 (23.5)	4.64	1.11–19.32				
No	26 (76.5)	1					
Extraintestinal manifestation				.030			.339
Present	4 (11.8)	4.98	1.17–21.29				
Absent	30 (88.2)	1					
Corticosteroid				.606			
Yes	2 (5.9)						
No	32 (94.1)						
Mesalazine				.888			
Yes	8 (23.5)						
No	26 (76.5)						
Immunosuppresion				.317			
Yes	7 (20.6)						
No	27 (79.4)						
Intraoperative blood transfusion				.606			
Yes	3 (8.8)						
No	31 (91.2)						
Intensive care				.396			
Yes	8 (23.5)						
No	26 (76.5)						
≥ Clavien-Dindo I				.513			
Yes	14 (41.2)						
No	20 (58.8						
≥ Clavien-Dindo IIIb				.940			
Yes	7 (20.6)						
No	27 (79.4)						
SSI				.967			
Yes	5 (14.7)						
No	28 (82.4)						
Seroma				.995			
Yes	9 (26.5)						
No	25 (73.5)						
Relaparotomy				.967			
Yes	5 (14.7)						
No	29 (85.3)						

Various parameters are associated with major and general postoperative morbidity. Hazard ratios are shown for statistically significant variables. *ASA* American society of anesthesiologist’s classification, *BMI* body mass index, *CVD* cardiovascular disease, *CKD* chronic kidney disease, *DM* diabetes mellitus, *CRD* chronic respiratory disease, *IBD* inflammatory bowel disease

## Discussion

In this study, we analyzed the perioperative outcomes after OVHR in patients with IBD and compared them to a cohort of non-IBD patients. Furthermore, we investigated the long-term outcome of IH repair in IBD patients to identify risk factors for hernia recurrence in this specific cohort. We here provide evidence that patients with IBD showed a significant higher rate of major complications (Clavien-Dindo ≥ 3b) after OVHR, while the incidence of overall complications (Clavien Dindo ≥ 1) was not significantly elevated compared to a non-IBD group. Additionally, a multivariable binary logistic regression model identified history of malignancy and the necessity of an intensive care stay as an independent risk factor for the occurrence of any complication (Clavien Dindo ≥ 1) and the presence of an IBD as the only significant risk factors for the occurrence of major complications (Clavien-Dindo ≥ 3b) which supports the observation from the comparison of the postoperative course of both groups. Analysis of the long-term outcome displayed hernia recurrence in 26.5% after a median follow-up of 36 months and identified the presence of UC as the single independent predictor of hernia recurrence.

Currently, only a few studies investigate the postoperative course after OVHR in IBD patients which are commonly based on heterogeneous patient cohorts. A study by Heiman et al. includes 170 patients over a long period of 38 years from 1976 to 2014 comprises a heterogeneous cohort with a wide range of surgical techniques and mesh types and positions [6]. Also, synthetic mesh implant was used in only 50.6% of the patients. Further, in 59% cases the mesh was then positioned in onlay position which is not considered state of the art by current standards [7]. From our point of view, these heterogeneous results are hardly comparable and the techniques are partly no longer established in clinical use. In the complicated situation of an incisional hernia in IBD patients, retromuscular mesh augmentation using a sublay plastic is the preferred treatment method, which provides an efficient mesh integration [8, 9]. In particular, the sublay position allows to avoid direct contact between the prosthesis and the underlying intestine and thus reduces the risk of adhesions, mesh erosion and the development of intestinal fistulas and obstructions, especially in patients with CD [10, 11]. Analogous to our study, Maman et al. report the experience of 59 patients who have been treated with OVHR using a sublay mesh of whom 38 have undergone primary surgery for IBD [12]. Major complications occurred in 15.2% of all cases with no distinction between IBD and non-IBD. This is consistent with the incidence of major complications observed in our cohort of 5.5% in the non-IBD and 20.6% in the IBD group. This difference and the fact that the presence of IBD in multivariate analysis was the single independent risk factor for the occurrence of major complications shows that IBD patients have a significantly increased surgical risk in OVHR and must be treated with caution. This emphasizes the importance of these patients being managed by a specialist during preoperative and postoperative follow-up in accordance with current guidelines [13, 14].

An often-described therapy option in potential contaminated situations is the implantation of a biological mesh [15–17]. A study by Wang et al. compared the outcome of OVHR with synthetic and biological mesh in 38 IBD-patients and found a significantly inferior short term outcome in the biological mesh group [2], which contradicts the perception concerning the superiority of the use of biologic mesh [18–20]. We share the opinion that the implantation of a synthetic mesh is possible with acceptable complication rates even in complex, potentially contaminated cases like IBD patients, which is in line with the results of Carbonell et al., who demonstrated favorable complication and recurrence rates associated with the use of synthetic mesh in contaminated situation [21]. During follow-up of the IBD cohort, we observed hernia recurrence in 26.5% of cases. This high recurrence rate, however, corresponds to the published data of 25% after 5 and 32% after 10 years for mesh-based OVHR [22–24], as well as the recurrence rate from the German hernia registry of 22% [25]. However, a heterogeneous range of different mesh types and materials is used for the OVHR in the reported studies. This retrospective analysis by Sanchez-Arteaga et al. investigates patients with OVHR who underwent emergency surgery involving only PVDF meshes [26]. They report a high one-year recurrence rate of 19% which is also comparable to our results, considering they analyzed solely emergency procedures. To our knowledge distribution of PVDF mesh to North America is not yet possible, which certainly limits the interest from this region and can explain the heterogeneous study situation on this mesh type so far.

Univariable analysis identified the presence of UC, history of > 1 bowel resection, and extraintestinal manifestation as risk factors for hernia recurrence after OVHR, while in the multivariable analysis only UC showed a statistically significant association. However, the lack of correlation with > 1 bowel resection (P = 0.096) might be a result of a lack of statistical power related to the small sample size as Heiman et al. have already shown this correlation [6]. The significantly higher recurrence rate after UC compared to CD has not been reported up to now. However, a selection bias due to the primary surgical technique could be an explanation. Similar to many other centers, laparoscopic proctocolectomy is also becoming the gold standard for the surgical treatment of UC in our surgical deparment [27], so that patients, who had conventional open surgery with a higher risk of IH, may have been the more complex cases or patients with a complicated postoperative course, which is not reflected in our data. The previously cited, only available study on this topic by Heiman et al. found no difference in the recurrence rate between UC and CD [6].

This study reviews a cohort of IBD patients who underwent OVHR in a homogenous technique. In addition, it is the first study to compare these results with a cohort of non-IBD patients undergoing OVHR with sublay mesh augmentation which minimizes bias of the results by technical variation. However, our analysis has certainly limitations that need to be discussed. First, the results are based on single-center cohort analyzed in a retrospective fashion with a limited number of patients in the IBD group; therefore, it is underpowered to reach a definitive conclusion and warrants further confirmation from other groups. Also, surgical technique has shifted to the laparoscopic approach for therapy of IBD, which may result in a significantly lower incidence of IH in the future.

## Conclusion

In conclusion this study shows that OVHR is feasible in patients with IBD. However, an intensive preoperative assessment should be carried out as there is a significantly increased risk of major complications compared to non-IBD patients.

## Data Availability

The datasets generated and analysed during the current study are not publicly available due to the local privacy policy on clinical data but are available from the corresponding author on reasonable request.

## Abbreviations

ASA: American society of anesthesiologists
BMI\: Body-mass-index
CD: Crohn's disease
CKD: Chronic kidney disease
cmCT: Contrast-material enhanced computed tomography
CRD: Chronic respiratory disease
CVD: Cardiovascular disease
DM: Diabetes mellitus
IBD: Inflammatory bowel disease
IH: Incisional hernia
OVHR: Open ventral hernia repair
SSI: Surgical site infect
UC: Ulcerative colitis
